# Correlates of Poor Quality of Life Among Older People Living With HIV: A Cross‐Sectional Analysis of the Tanzania HIV and Aging Cohort Study

**DOI:** 10.1002/jia2.70163

**Published:** 2026-07-17

**Authors:** Theresia A. Ottaru, Christopher Mbotwa, Dannielle Grayer, Joan Rugemalila, Lisa R. Hirschhorn, Sylvia Kaaya, Grayson Nyamsogoro, Rosemary Kiboma, Herieth Mboya, Irene Mageni, Pilly Chillo, Edith Tarimo, Mary Clare Masters, Bethann Conover, Claudia Hawkins

**Affiliations:** ^1^ Department of Epidemiology and Biostatistics Muhimbili University of Health and Allied Sciences Dar es Salaam Tanzania; ^2^ Mbeya College of Health and Allied Sciences University of Dar es Salaam Mbeya Tanzania; ^3^ Department of Medicine, Feinberg School of Medicine Northwestern University Chicago Illinois USA; ^4^ Department of Internal Medicine Muhimbili National Hospital Dar es Salaam Tanzania; ^5^ Department of Medical Social Sciences, Feinberg School of Medicine Northwestern University Chicago Illinois USA; ^6^ Robert J. Havey Institute for Global Health, Feinberg School of Medicine Northwestern University Chicago Illinois USA; ^7^ Department of Psychiatry and Mental Health Muhimbili University of Health and Allied Sciences Dar es Salaam Tanzania; ^8^ Department of Environmental Health and Ecological Sciences Ifakara Health Institute Dar es Salaam Tanzania; ^9^ Department of Internal Medicine Muhimbili University of Health and Allied Sciences Dar es Salaam Tanzania; ^10^ Department of Nursing Management Muhimbili University of Health and Allied Sciences Dar es Salaam Tanzania; ^11^ Albert Einstein College of Medicine, Montefiore Medical Center Bronx New York USA

**Keywords:** ageing, geriatric syndromes, HIV, quality of life, Tanzania

## Abstract

**Introduction:**

Older people living with HIV (PLH) have a high multimorbidity burden that may impair quality of life (QOL). This study assessed the burden of geriatric syndromes and medical comorbidities and their association with QOL among older PLH in urban Tanzania. These results are important to understand the growing complexities of care needs in this population and inform future strategies.

**Methods:**

We conducted a cross‐sectional analysis of baseline data from the Tanzania HIV and Aging Longitudinal Cohort Study (THALCS). Eligible participants (age ≥50 years, on anti‐retroviral therapy [ART] for ≥3 years) were recruited between April and July 2024 from seven HIV care and treatment centres. Participants were excluded if pregnant or unable to consent. QOL was assessed using the WHOQOL‐HIV‐BREF, a 31‐item tool in six domains. Domain scores were converted to a 0–100 scale, and the overall QOL score was calculated. Geriatric syndromes and medical comorbidities were assessed using standard tools. Multivariable linear regression examined the associations, adjusting for sociodemographic and HIV‐related factors.

**Results:**

Among the 400 participants (median age: 57 [IQR: 53−63] years; 50% females; ART duration: 10−19 years [60.3%]), the majority were on first‐line dolutegravir‐based ART (83.5%) with undetectable viral load (78.5%). The most common medical comorbidities were dyslipidaemia (80.2%), hypertension (54.5%) and overweight/obese (50.8%). Frailty (13.8%) and pre‐frailty (48.3%) were observed. Median QOL score was 75.4 (IQR: 66.3−83.5), highest in physical health and spirituality domains (87.5 [IQR: 75−100] and 87.5 [IQR: 68.7−93.7]) and lowest in environmental domain 62.5 (IQR: 50−75). Males had a higher median overall QOL score than females (76.8 [IQR: 67.9−83.7] vs. 72.9 [IQR: 65.2−83.4], *p* = 0.036). Pre‐frailty (β = −3.41, 95% CI: −5.64, −1.18, *p* = 0.003), mild depression (β = −7.88, 95% CI: −11.17, −4.60, *p*<0.001) and moderate/severe depression (β = −19.25, 95% CI: −24.67, −13.83, *p*<0.001) were associated with poorer QOL. Functional impairment showed a dose–response relationship with QOL. Increasing age (β = 0.18, 95% CI: 0.02, 0.34; *p* = 0.026) and higher income (>500,000 TZS) (β = 7.16, 95% CI: 3.10, 11.21; *p* = 0.001) were associated with better QOL.

**Conclusions:**

A high prevalence of geriatric syndromes and medical comorbidities was observed among older PLH in Tanzania, several of which were negatively associated with QOL. These findings underscore the need to integrate holistic care models in HIV programmes for older PLH.

## 1 Introduction

HIV programmes in sub‐Saharan Africa (SSA) are among the most successful healthcare delivery initiatives globally [1, 2]. Over the past two decades, there has been a substantial improvement in access to effective anti‐retroviral therapy (ART) [3, 4], and, as a result, improved life expectancy and an increasing number of people living with HIV (PLH) are living into older age [5, 6]. In Tanzania, approximately 31% of the 1.7 million PLH are aged 50 years and above, and it is estimated that this population will grow by 25% by 2040 [7, 8].

Ageing is a biological process characterized by molecular and cellular damage, which increases the risk of multimorbidity, including geriatric syndromes such as frailty and cognitive impairment, and medical comorbidities such as cardiovascular diseases, renal dysfunction and diabetes [9, 10]. HIV‐associated chronic inflammation and immune activation accelerate and accentuate the ageing process, further increasing the risk of multimorbidity in this population [11, 12, 13]. Complications related to ageing and multimorbidity tend to appear at an earlier age and are often more severe among older PLH compared to those without HIV [14].

Studies from SSA report a substantial burden of multimorbidity among older PLH; 34.2% have hypertension, 7.8% have diabetes and 3.9% have heart disease [15]. Approximately 36% of PLH ≥50 and 54.1% of PLH ≥60 in the region are reported to have at least one age‐related medical comorbidity [15, 16]. Data on geriatric syndromes among older PLH in SSA remain limited [17]. The World Health Organization (WHO) defines geriatric syndromes as multifactorial clinical conditions in older adults, involving multiple organ systems and not attributable to specific disease categories [18]. Existing studies from SSA estimate that 3%−15% of older PLH are frail and 30%–75% are prefrail [19, 20, 21, 22, 23], while approximately half have mild cognitive impairment, and 6% meet criteria for dementia [24]. Functional decline and mental health disorders are also common, with more than 10% reporting functional decline [25] and experiencing severe depressive symptoms [26].

Declines in physical and cognitive functioning related to geriatric syndromes and symptoms related to medical comorbidities such as pain and fatigue negatively affect the quality of life (QOL) of older PLH [14, 27, 28, 29]. This impact is especially pronounced among older PLH in SSA who face social and economic vulnerabilities, such as poverty and poor living conditions [30]. These challenges, coupled with the limited availability of age‐appropriate healthcare services [31], hinder older PLHs’ ability to access and maintain care [32].

Few studies have examined the burden and impact of medical comorbidities and geriatric syndromes on QOL among older PLH in Tanzania. We assessed the prevalence of geriatric syndromes (frailty, functional and cognitive impairment, and depression) and medical comorbidities (hypertension, diabetes, dyslipidaemia, renal dysfunction and obesity) and their association with QOL among PLH ≥50 years in urban Tanzania. As the population of older PLH continues to grow, the results of this study will be important in guiding the development of tailored healthcare interventions that will improve QOL of older PLH in this region.

## 2 Methods

### 2.1 Study Design, Setting and Population

We conducted a cross‐sectional analysis of baseline data from the Tanzania HIV and Aging Longitudinal Cohort Study (THALCS), an established cohort of 400 PLH aged ≥50 years in Dar es Salaam, Tanzania. THALCS is a multisite, multidisciplinary longitudinal research platform on HIV and ageing in Dar es Salaam, Tanzania, aimed at generating local evidence to guide optimal care for older PLH. Participants were enrolled between April and July 2024 from seven high‐volume HIV care and treatment centres (CTCs) in Dar es Salaam, defined as centres serving ≥1500 PLH. These CTCs were randomly selected from a list of high‐volume centres across the region, representing five Dar es Salaam districts and three levels of Tanzania's healthcare delivery system (primary, secondary and tertiary level).

At each site, participants were recruited using systematic random sampling. A proportional to size approach was used to allocate the sample across the selected HIV CTCs. At each site, the sample was stratified by sex (equal sample size to males and females). Eligible participants were PLH aged ≥50 years who had been on ART for ≥3 years and enrolled in one of the seven HIV CTCs for ≥12 months. Participants were excluded if they were pregnant during the study period or unable to consent.

#### 2.1.1 HIV Care and Treatment in Tanzania

HIV CTCs in Tanzania are outpatient clinics that provide HIV care, including HIV prevention, testing and treatment, including treatment of related co‐infections. Depending on how stable a patient is on ART, appointments to CTCs can range from monthly to every 6 months. National guidelines [33] recommend that CTCs provide patient education on lifestyle modification, and screen for and/or manage medical comorbidities (hypertension, diabetes, obesity, dyslipidaemia and renal disease). When medical comorbidities cannot be managed within the HIV CTCs, healthcare providers are required to refer PLH to medical outpatient or specialized clinics for appropriate management. Implementation of these guideline recommendations varies across CTCs depending on the level of support from implementing partners, CTC providers’ expertise and the laboratory capacity of the health facility. Screening for geriatric syndromes, such as frailty, cognitive impairment and functional decline, is not part of routine care at CTCs in Tanzania.

### 2.2 Data Collection

An interviewer‐administered questionnaire programmed in REDCap was used to collect self‐reported data on sociodemographic characteristics (age, sex, marital status, education, employment status, income and insurance status), QOL, social support, history of medical comorbidities (hypertension, diabetes, dyslipidaemia, renal disease), number of medications used and geriatric syndromes (depression, frailty, functional and cognitive impairment).

For the assessment of medical comorbidities and geriatric syndromes, participants underwent blood pressure (BP), height and weight (for body mass index [BMI]) measurements and physical assessments measuring walking speed and hand grip strength to calculate the frailty score. BP was measured using a calibrated portable battery‐operated automatic BP cuff (Lifesource UA‐767 PV), following American Heart Association (AHA) recommendations [34]. Height and weight for BMI were measured using standard calibrated height/weight measuring devices (stadiometer and digital scale), and grip strength was measured using a Jamar smart hand dynamometer. A single vial (approx. 15cc) of blood was also drawn for fasting blood glucose (FBG), lipid profile, and serum creatinine and 5cc of urine was collected to assess for proteinuria. FBG was measured using Food and Drug Administration (FDA)‐approved, Accu‐Check Guide glucose monitor. Lipid profile and serum creatinine were measured using COBAS INTEGRA 400 plus by Cobas Roche Diagnostics. Urine tests for proteinuria and albuminuria were conducted using CYANStrip Mini (Cod. CY011S02) machine.

We reviewed medical records to obtain HIV‐related clinical data, including years living with HIV, HIV viral load (VL), duration on and type of ART. Participants found to have elevated values of BP (mean systolic BP ≥140 mmHg and/or mean diastolic BP ≥90 mmHg), FBG ≥7 mmol/L, with dyslipidaemia and depressive symptoms were linked to the medical outpatient clinic at the respective health facility for management.

## 3 Measures

We assessed QOL using the Swahili version of WHOQOL‐HIV‐BREF, 2012 revision [35]. WHOQOL‐HIV‐BREF contains 31 items in six domains: physical health (four items), psychological health (five items), level of independence (four items), social relationships (four items), environmental health (eight items), spirituality (four items). Two items measure participants’ perception of general QOL and health status [35]. In questions for which a higher score did not mean better QOL, the responses were first inversely scored and then calculated. The total score was computed for each of the domains and converted to a 0−100 scale. The overall QOL score was computed as the average of the scores of the six domains. The WHOQOL‐HIV‐BREF indicated a good internal consistency with a Cronbach alpha of 0.86.

*Geriatric comorbidities*, including frailty, functional and cognitive impairment, depression and social support, were assessed in this study. Table 1 provides a detailed summary of the assessment tools used and the operational definitions for each variable.

**TABLE 1:** Assessment tools and operational definitions for geriatric comorbidities.

Comorbidity	Tool	Items	Operational definition
Frailty	Fried's Frailty phenotype score [41]	Five items: (1) unintentional weight loss, (2) poor endurance and energy, (3) low physical activity, (4) slowness (walking speed over 6 m) and (5) weakness (grip strength) Outcome: Presence of ≥ 3 of the following: weight loss, exhaustion, low physical activity, weak grip strength and slow walking speed. For each of the items, the individual was scored 1 if present and 0 if absent. Overall score ranges from 0 to 5, with higher scores indicating more frailty.	*No frail*—score of 0 *Pre‐frail*—score 1−2 *frail*—score ≥3
Cognitive functioning	Brief community screening interview for dementia (CSI‐D) [42]	Eight items adapted from the CSI‐D instrument that assessed: memory (recall and registration) (1 item), language (expression and naming) (3 items), orientation (to place and time) (4 items). Overall score ranges from 0 to 9, with higher scores indicating better cognitive function	*No cognitive impairment*: 7−9 *Mild cognitive impairment*: 5−6 *Dementia*: ≤4
Functional impairment	World Health Organization Disability Assessment Schedule 2.0 (WHODAS 2.0) [43]	Twelve items adapted from the WHODAS 2.0 that assess level of difficulty experienced in the past 30 days in (1) moving around, (2) concentrating and remembering things, (3) learning new task, (4) standing for long time, (5) bathing/washing, (6) getting dressed, (7) carrying things, (8) eating, (9) getting up from lying, (10) using toilet, (11) using public transportation and (12) going out.	*Functional impairment categories* *None* *1*−*3 functional difficulties* *4*−*6 functional difficulties* *>6 functional difficulties*
Social support	Lubben Social Network Scale [44]	Six‐item scale that assesses social engagement, including with family and friends. Scores range from 0 to 30, and higher scores indicate more social engagement.	*At risk of social isolation*—score<12
Depression	PHQ 9 [45]	Nine items that assess the experience of depressive symptoms in the past 2 weeks. Each item had four possible responses, ranging from 0 “Not at all,” 1 “Several days,” 2 “More than half a day” and 3 “Nearly every day.” The total score for all items ranges from 0 to 27.	*None or minimal*: <5 *Mild depression*: 5−9 *Moderate depression*: 10−14 *Moderately severe*: 15−19 Severe: 20−27

For *Medical comorbidities*, participants were classified as people with hypertension if the mean systolic BP is ≥140 mmHg and/or the mean diastolic BP is ≥90 mmHg, calculated from an average of three values taken at least 5 min apart, or if they self‐reported current use of antihypertensive medication. Participants were classified as people with diabetes if FBG was ≥7 mmol/L or if they self‐reported current use of diabetes medication [36].

Participants were classified as having dyslipidaemia if they had low‐density lipoprotein (LDL) cholesterol: >2.6 mmol/L or Triglycerides: >1.7 mmol/L or Total cholesterol: >5.2 mmol/L or high‐density lipoprotein (HDL): <1 mmol/L regardless of sex [37]. Estimated glomerular filtration rate (eGFR) was calculated using the 2021 CKD‐EPI equation. Renal dysfunction was defined as an eGFR of <60 mL/min/1.73 m^2^ (with or without proteinuria) [38]. To assess the number of medications used, participants were asked how many prescribed medications they took per day (excluding ARTs), and were classified into two groups (<2 vs. ≥2 medications) [39]. Assessment of health behaviours including tobacco and alcohol use followed the WHO STEPS NCD risk assessment tool [40].

### 3.1 Data Analysis

All data were stored in REDCap and were then imported into STATA version 18 (STATA Corp Inc., TX, USA) for analyses. Descriptive statistics disaggregated by sex were used to summarize characteristics of the study population; means and standard deviations for continuous variables and frequencies and percentages for categorical variables. Differences between males and females were assessed using the Mood's median test for continuous variables (age, overall QoL score and domain‐specific QoL scores), and the chi‐square test for categorical variables. A test for normality was conducted using the Shapiro−Wilk test (*p* = 0.20) and the Kolmogorov−Smirnov test (*p* = 0.471). In addition, the distribution of regression residuals was assessed through visual inspection of histograms, Q‐Q plots and P‐P plots, which indicated no deviation from normality. After confirming no significant violation of model assumptions for linear regression, we applied multivariable (MV) linear regression to identify medical comorbidities (hypertension, diabetes, dyslipidaemia, renal dysfunction and BMI) and geriatric comorbidities (frailty, cognition, functional impairment, depression, number of medications used and social support) associated with QOL, adjusting for sociodemographic factors (age, sex, education level, income and health insurance status) and HIV‐related characteristics (duration on ART and VL suppression). We found a moderate correlation (*r* = 0.53) between PHQ‐9 scores for depression and the psychological domain. We further explored effect modification by sex for geriatric syndromes and medical comorbidities and included the significant interaction in the MV. We also conducted domain‐specific MV linear regression to identify correlates of QOL domain‐specific scores. A *p*‐value <0.05 was considered significant.

### 3.2 Ethical Consideration

The study was approved by the ethics committee of Muhimbili University of Health and Allied Sciences (MUHAS‐REC‐0302024‐2104), National Institute for Medical Research (NIMR/HQ/R.8a/Vol.IX/4880) and Northwestern University (STU00221493). We also obtained clearance to conduct the study from the President's Office Regional Administration and Local Government (PO‐RALG) and the selected HIV CTCs. Written informed consent was obtained from all study participants after they had been thoroughly introduced to the study. Voluntary participation, anonymity and confidentiality were maintained throughout the study.

## 4 Results

### 4.1 Baseline Characteristics of Older PLH in Urban Tanzania

A total of 400 PLH aged ≥50 years, median age 57 (IQR: 53−63) years, 50% males, were included in this analysis. The majority of the participants were aged between 50 and 59 years (61.8%); 8% were aged ≥70 years (Table 2). Males were more likely than females to be in a marital union (63% vs. 20%, *p*<0.001), to be employed or self‐employed (80.9% vs. 67%, *p* = 0.002) and to report higher income (>100,000 TZS) (74% vs. 42.5%, *p*<0.001).

**TABLE 2:** Baseline characteristics of older PLH in urban Tanzania by sex.

Variable	All (*N* = 400)	Female (*N* = 200)	Male (*N* = 200)	*p*‐value
	*n* (%)	*n* (%)	*n* (%)	
Age in years (Median [IQR])	57 (53−63)	56 (53−61)	58 (54−64)	0.109
50−59	247 (61.8)	133 (66.5)	114 (57.0)	
60−69	121 (30.2)	51 (25.5)	70 (35.0)	
70+	32 (8.0)	16 (8.0)	16 (8.0)	
Marital status				<0.001
Currently in marital union	166 (41.5)	40 (20.0)	126 (63.0)	
Currently not in marital union	234 (58.5)	160 (80.0)	74 (37.0)	
Education level				0.059
No formal education	55 (13.8)	35 (17.5)	20 (10.0)	
Primary	264 (66.0)	130 (65.0)	134 (67.0)	
Secondary+	81 (20.2)	35 (17.5)	46 (23.0)	
Primary occupation				0.002
Retired or unemployed	104 (26.1)	66 (33.0)	38 (19.1)	
Employed or self‐employed	295 (73.9)	134 (67.0)	161 (80.9)	
Estimated monthly income (TZS)				<0.001
< 100,000	167 (41.8)	115 (57.5)	52 (26.0)	
100,000−500,000	195 (48.8)	74 (37.0)	121 (60.5)	
500,001−1,000,000	25 (6.2)	8 (4.0)	17 (8.5)	
>1,000,000	13 (3.2)	3 (1.5)	10 (5.0)	
Has health insurance (yes)	82 (20.5)	40 (20.0)	42 (21.0)	0.804
Drink alcohol (yes)^a^	112 (28.0)	48 (24.0)	64 (32.0)	0.075
Smoking cigarette (yes)^b^	28 (7.0)	1 (0.5)	27 (13.5)	<0.001

^a^Drinking alcohol: current—in the past 30 days versus never or past drinker.

^b^Smoking cigarette: current—in the past 30 days versus never or past smoker.

### 4.2 Distribution of Comorbidities and HIV‐Related Characteristics Among Older PLH in Urban Tanzania

Over half of the participants had been living with HIV for 10−19 years (57.9%), had undetectable VL (<50 copies/mL) (78.5%) and were on a first‐line dolutegravir (DTG)‐based ART regimen (83.5%). Frailty was present in 13.8% of participants, while nearly half (48.3%) were classified as prefrail. The most prevalent medical comorbidity was dyslipidaemia (80.2%), and hypertension was present in over half (Table 3). Females had a significantly higher prevalence of being at risk of social isolation (33.5% vs. 15%, *p*<0.001), having ≥1 functional difficulty (41.5% vs. 33%, *p* = 0.002), renal impairment (19.5% vs. 12%, *p* = 0.040) and overweight/obese (59.5% vs. 42%, *p*<0.001) compared with males.

**TABLE 3:** Distribution of geriatric syndromes, medical comorbidities and HIV‐related characteristics among older PLH in urban Tanzania by sex.

Variable	All (*N* = 400)	Female (*N* = 200)	Male (*N* = 200)	*p*‐value
	*n* (%)	*n* (%)	*n* (%)	
**Geriatric syndromes**				
Frailty				0.072
No frail	145 (36.3)	66 (33.0)	79 (39.5)	
Pre‐frail	193 (48.3)	96 (48.0)	97 (48.5)	
Frail	55 (13.8)	35 (17.5)	20 (10.0)	
Cognition				0.133
No cognitive impairment	377 (94.2)	185 (92.5)	192 (96.0)	
Mild cognitive impairment/dementia	23 (5.8)	15 (7.5)	8 (4.0)	
Functional impairment				0.002
None	234 (58.5)	100 (50.0)	134 (67.0)	
1−3	106 (26.5)	61 (30.5)	45 (22.5)	
4−6	38 (9.5)	22 (11.0)	16 (8.0)	
>6	22 (5.5)	17 (8.5)	5 (2.5)	
At risk of social isolation	97 (24.2)	67 (33.5)	30 (15.0)	<0.001
Depression (PHQ‐9)				0.184
None or minimal (0−4)	291 (72.8)	138 (69.0)	153 (76.5)	
Mild (5−9)	76 (19.0)	45 (22.5)	31 (15.5)	
Moderate/moderately severe (10−19)	33 (8.2)	17 (8.5)	16 (8.0)	
**Medical comorbidities**				
Hypertension^a^	218 (54.5)	106 (53.0)	112 (56.0)	0.547
Diabetes^b^	47 (11.8)	25 (12.5)	22 (11.0)	0.475
Dyslipidaemia^c^				0.792
Normal	67 (16.8)	35 (17.5)	32 (16.0)	
Dyslipidaemia	321 (80.2)	162 (81.0)	159 (79.5)	
Renal dysfunction^d^	63 (15.8)	39 (19.5)	24 (12.0)	0.040
Number of medications used				0.067
0−1	349 (87.3)	168 (84.0)	181 (90.5)	
2+	50 (12.5)	31 (15.5)	19 (9.5)	
BMI				<0.001
Underweight (<18.5)	25 (6.3)	10 (5.0)	15 (7.5)	
Normal (18.5−24.9)	168 (42.4)	69 (34.5)	99 (49.5)	
Overweight (25−29.9)	110 (27.8)	47 (23.5)	63 (31.5)	
Obese (30+)	93 (23.5)	72 (36.0)	21 (10.5)	
**HIV‐related characteristics**				
Years lived with HIV				0.934
<10	141 (35.3)	72 (36.0)	69 (34.7)	
10−19	231 (57.9)	114 (57.0)	117 (58.8)	
≥20	27 (6.8)	14 (7.0)	13 (6.5)	
Duration since in ART				0.676
<10	148 (37.2)	77 (38.5)	71 (35.9)	
10−19	240 (60.3)	117 (58.5)	123 (62.1)	
≥20	10 (2.5)	6 (3.0)	4 (2.0)	
Current ART regimen				0.611
TDF + 3TC + DTG (TLD)	333 (83.5)	171 (85.5)	162 (81.4)	
Other	66 (16.5)	29 (14.5)	37 (18.5)	
Undetectable viral load (< 50 copies/mL)	314 (78.5)	159 (79.5)	155(77.5)	0.998
Adherent to ART in the past 30 days	324 (81.0)	164 (82.0)	160 (80.0)	0.610
History of opportunistic infections	199 (49.8)	96 (48.0)	103 (51.5)	0.484

Abbreviations: DTG, dolutegravir; 3TC, lamivudine; TDF, tenofovir; TLD, tenofovir, lamivudine and dolutegravir.

^a^Hypertension: SBP≥140 mmHg or DBP≥90 mmHg or on antihypertensive medication.

^b^Diabetes: FBG≥7 mmol/L or on antidiabetic medication.

^c^Dyslipidaemia: At least one of these: LDL cholesterol: > 2.6 mmol/L or Triglycerides: > 1.7 mmol/L or Total cholesterol: > 5.2 mmol/L or HDL: < 1 mmol/L regardless of sex.

^d^Renal dysfunction: eGFR<60 mL/min/1.73 m^2^.

Adherent to ART: Self‐reported of no missed pills in the past 30 days.

BMI has four missing (two males, two females), frailty has seven missing (four males, three females), viral load has nine missing (seven males, two females), dyslipidaemia has 12 missing (nine males, three females). Number of medications used has one missing for male.

### 4.3 Distribution of QOL Scores (Overall and Domain‐Specific) Among Older PLH in Urban Tanzania

**FIGURE 1:**
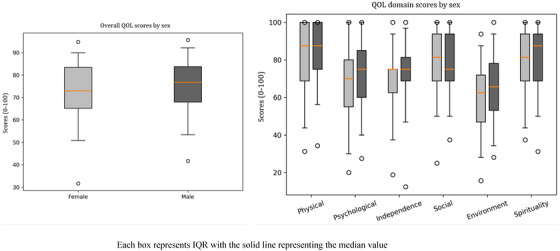
Distribution of QOL scores (overall and domain‐specific) among older PLH in urban Tanzania by sex.

The overall median QOL score was 75.4 (IQR: 66.3–83.5). The physical health and spirituality domains had the highest median scores, both at 87.5 (IQR: 75–100 and 68.7–93.7, respectively), followed by the social relationships domain at 81.3 (IQR: 68.7–93.7). The environmental domain had the lowest median score of 62.5 (IQR: 50–75). For the general QOL questions, only 36 (9%) of all 400 participants rated their QOL as good or very good. However, 280 (70%) reported being either satisfied or very satisfied with their health. Males had higher median overall QOL score than females (76.8 [IQR: 67.9–83.7] vs. 72.9 [IQR: 65.2–83.4], *p* = 0.036) and lower median score in the social relationship domain (75 [IQR: 68.8–93.8] vs. 81.3 [IQR: 68.8–93.8], *p* = 0.026) (Figure 1).

### 4.4 Association Between Geriatric Syndromes and Medical Comorbidities and QOL Among Older PLH in Urban Tanzania

In MV analysis (Table 4), adjusting for age, sex, education level, income, health insurance, ART duration, HIV VL and medical comorbidities, several geriatric conditions were associated with QOL. Functional impairment showed a dose–response relationship with QOL, with progressively greater reductions observed as the number of difficulties increased.

**TABLE 4:** Univariate and multivariate regression analyses for medical comorbidities and geriatric syndromes associated with QOL.

Variables	Univariate analyses		Multivariable analyses^a^	
	β (95% CI)	*p*‐value	β (95% CI)	*p*‐value
**Geriatric conditions**				
Frailty				
No frail	Ref.	Ref.		
Pre‐frail	−6.86 (−9.26, −4.45)	<0.001	−3.41 (−5.64, −1.18)	0.003
Frail	−15.33 (−18.79, −11.86)	<0.001	−2.06 (−5.99 −1.86)	0.302
Cognition				
No cognitive impairment	Ref.	Ref.		
Mild cognitive/dementia	−1.17 (−6.34, 4.00)	0.657	0.19 (−3.92, 4.32)	0.925
Functional impairment				
None	Ref	Ref		
1−3	−8.20 (−10.60, −5.81)	<0.001	−6.36 (−8.79, −3.94)	<0.001
4−6	−13.72 (−17.30, −10.15)	<0.001	−8.44 (−12.32, −4.55)	<0.001
>6	−23.02 (−27.57, −18.46)	<0.001	−13.94 (−19.44, −8.44)	<0.001
At risk of social isolation	−4.63 (−7.40, −1.85)	0.001	−0.19 (−2.53, 2.15)	0.875
Depression (PHQ‐9)				
None or minimal (0−4)	Ref.	Ref.		
Mild (5−9)	−11.37 (−14.01, −8.73)	<0.001	−7.88 (−11.17, −4.60)	<0.001
Moderate/moderately severe (10−19)	−19.42 (−23.19, −15.66)	<0.001	−19.25 (−24.67, −13.83)	<0.001
**Medical comorbidities**				
Hypertension^a^	−1.82 (−4.24, 0.59)	0.138	−1.75 (−3.86, 0.36)	0.103
Diabetes^b^	−1.92 (−5.66, 1.81)	0.312	2.16 (−0.94, 5.26)	0.172
Renal dysfunction^c^	−0.66 (−3.96, 2.65)	0.695	0.48 (−2.22, 3.19)	0.726
Dyslipidaemia^b^	−2.25 (−5.48, 0.98)	0.172	−1.51 (−4.07, 1.05)	0.247
Number of medications used				
0−1	Ref.	Ref.		
≥2	−8.54 (−12.08, −4.99)	<0.001	−3.47 (−6.89, −0.05)	0.047
BMI (kg/m^2^)				
Normal	Ref.	Ref.		
Underweight	−5.96 (−11.07, −0.85)	0.022	−3.37 (−7.59, 0.85)	0.117
Overweight	−2.39 (−5.31, 0.53)	0.109	−0.92 (−3.27, 1.43)	0.422
Obese	−3.73 (−6.82, −0.65)	0.018	−0.85 (−3.56, 1.86)	0.537
**Adjusted confounders**				
Sex				
Female	Ref.	Ref.		
Male	2.81 (0.42, 5.20)	0.021	−1.69 (−4.15, 0.76)	0.176
Interaction between sex and depression				
Female*None or minimal depression	Ref.		Ref.	
Male*Mild depression	−1.82 (−7.13, 3.50)	0.502	−1.01 (−5.94, 3.91)	0.685
Male*moderate/Moderately severe depression	7.94 (0.46, 15.42)	0.037	7.71 (0.53, 14.89)	0.035
Age (in years)	−0.07 (−0.24, 0.11)	0.459	0.18 (0.02, 0.34)	0.026
Education level				
No formal education	Ref	Ref		
Primary	1.85 (−1.69, 5.38)	0.305	−0.81 (−3.78, 2.16)	0.591
Secondary+	5.62 (1.45, 9.79)	0.008	−0.96 (−4.71, 2.80)	0.617
Estimated monthly income (TZS)				
< 100,000				
100,000−500,000	4.67 (2.20, 7.13)	<0.001	2.12 (−0.09, 4.33)	0.060
>500,000	9.70 (5.50, 13.90)	<0.001	7.16 (3.10, 11.21)	0.001
Has health insurance	0.025 (−2.96, 3.01)	0.987	2.05 (−0.62, 4.72)	0.133
Duration since in ART				
<10	Ref			
10−19	−0.44 (−2.96, 2.09)	0.733	−0.82 (−2.79, 1.14)	0.409
≥20	−1.78 (−9.67, 6.11)	0.658	−1.00 (−7.34, 5.33)	0.756
Undetectable viral load (< 50 copies/mL)	−0.10 (−3.18, 2.98)	0.949	−0.85 (−3.20, 1.50)	0.478

^a^Hypertension: SBP≥140 mmHg or DBP≥90 mmHg or on antihypertensive medication.

^b^Diabetes: FBG≥7 mmol/L or on antidiabetic medication.

Dyslipidaemia: At least one of these: LDL cholesterol: > 2.6 mmol/L or Triglycerides: > 1.7 mmol/L or Total cholesterol: > 5.2 mmol/L or HDL: < 1 mmol/L regardless of sex.

^c^Renal dysfunction: eGFR<60 mL/min/1.73 m^2^.

Depression was independently associated with poorer QOL, with evidence of interaction by sex (Table 4). Moderate/moderately severe depression (vs. no/minimal depression) was associated with poorer QOL among females (β = −19.25, 95% CI: −24.67, −13.83, *p*<0.001), compared to males (β = −11.54, 95% CI: −16.75, −6.32, *p*<0.001), interaction β = 7.71, 95% CI: 0.53, 14.89, *p* = 0.035. Increasing age (β = 0.18, 95% CI: 0.02, 0.34; *p* = 0.026) and higher income (>500,000 TZS) (β = 7.16, 95% CI: 3.10, 11.21; *p* = 0.001) were associated with better QOL. In QOL domain‐specific MV regression analysis, pre‐frailty and frailty were associated with lower scores in physical and independence domains, while depression had a significant association with all QOL domains included in the analysis (Table S1).

## 5 Discussion

In this study of older PLH in urban Tanzania, a high prevalence of both geriatric syndromes and medical comorbidities was observed. Overall, older PLH in our study reported good QOL, with the highest scores reported in the physical health and spirituality domains and the lowest scores in the environmental domain. Geriatric syndromes (pre‐frailty, functional impairment and depression) were associated with poor QOL scores, while age and level of income were associated with better QOL scores.

Findings from this study add to a growing body of evidence highlighting the substantial burden of individual medical comorbidities and geriatric syndromes among older PLH in SSA [15, 46, 47, 48]. Despite this high burden, participants in our study reported a relatively high QOL consistent with findings from Tanzania (WHOQOL‐HIV‐BREF) [49], Uganda (WHOQOL‐OLD) [50] and Nigeria (WHOQL BREF) [51]. Compared to older people without HIV, literature from SSA suggests that older PLH in this setting may have better perceived QOL [52, 53, 54]. While the reported good QOL among older PLH in SSA may reflect the success of HIV programmes, other explanations should be considered. Survivorship bias may play a role, as people ≥50 years who remain engaged in HIV care may represent a healthier subgroup. In our study, increasing age was associated with better QOL scores. Another plausible explanation is that global definitions of QOL differ substantially in African settings, where social and family may carry greater significance [55]. This is further supported by our finding that only 9% of participants rated their QOL as “good” or “very good.” Developing contextually appropriate tools that more accurately reflect the lived experiences and priorities of older PLH in an African setting is essential for improving QOL assessments.

In our analysis of WHOQOL‐HIV‐BREF domains, the highest scores were observed in the physical health and spirituality domains, while the environmental domain had the lowest scores. This domain captures perceived access to basic needs, including financial resources and healthcare services [35]. The observed low score may be attributed to the high prevalence of functional impairment in this cohort, which likely limits access to essential services and worsens existing socioeconomic constraints. These findings highlight the need for care models that extend beyond healthcare facilities to address broader environmental challenges [55]. The relatively high spirituality domain scores align with previous regional research [56, 57], indicating that spiritual practices are key coping mechanisms among people living with chronic conditions in SSA. QOL interventions for older PLH may benefit from leveraging spiritual support systems.

Males reported better overall QOL scores, higher income levels and were more likely to be employed or self‐employed compared with females. Similar results have been reported among older PLH, where males reported better QOL than females, driven by socioeconomic disparities [58]. Higher income and employment can improve access to resources such as healthcare, nutrition and social participation, all of which contribute to better perceived wellbeing. Functional impairment was significantly more common among females, which may further contribute to their lower QOL.

Functional impairment, prefrailty and depression were associated with QOL, consistent with previous literature [29]. Both frailty and pre‐frailty have previously been associated with poorer QOL and higher all‐cause mortality among older PLH [50, 59]. The lack of a significant association between frailty and QOL in our study could be due to the small number of frail participants, who may represent a relatively healthier subset with better coping mechanisms and, therefore, better QOL. Also, pre‐frailty and frailty were associated with lower scores in the physical health and independence domains only, while depression was associated with lower scores across all domains included in the analysis, highlighting its strong association with overall wellbeing. While maintaining physical functioning and functional independence is critical for healthy ageing, our findings demonstrate that psychosocial health is also associated with wellbeing among older PLH. Notably, more than one in four participants in our study had depressive symptoms. Therefore, prioritizing routine depression screening and other mental health services in the design of holistic care for older PLH is essential to improving QOL. Depression was associated with poorer QOL in females than males, highlighting the need for gender‐tailored psychosocial interventions.

The bidirectional relationship between medical comorbidities and geriatric syndromes is well documented [60]. Evidence indicates that medical comorbidities can exacerbate geriatric syndromes, accelerate functional decline and increase the risk of loss of independence among older PLH [61, 62]. The substantial burden of individual medical comorbidities observed in our study population, despite showing no significant association with QOL, should not be overlooked. As PLH continue to age, an unaddressed burden of both geriatric syndromes and medical comorbidities can significantly impair QOL, making the integration of holistic care for multiple chronic conditions into HIV programmes imperative to healthy ageing in this population. In SSA, geriatric care remains largely absent from HIV programmes. Evidence from high‐income settings demonstrate that integrating geriatric care into HIV services is feasible and improves patient outcomes [63, 64, 65, 66]. In Tanzania, there has been progress through national guidelines and civil society advocacy. People aged ≥50 years were recently included as a special group in the guidelines for the management of HIV; however, provision of geriatric care has not been integrated into routine practice.

This study has some limitations. QOL was assessed using a validated and widely used tool (WHOQOL‐HIV‐BREF). As a structured tool, it may not fully explore the context‐specific complexities of lived experiences in this population. Future studies employing qualitative approaches may be valuable in providing a more in‐depth understanding of the QOL of older PLH based on their lived experiences. Our study consisted of a relatively young population of older PLH on ART (median age 57 [IQR: 53−63]), with the majority still employed or self‐employed. This sample may represent a healthier subgroup of older PLH, which could have contributed to the higher QOL scores obtained. Additionally, we recruited older PLH at HIV CTCs within an urban setting in Tanzania, potentially missing information from those not in HIV care and in rural areas. Rural populations may have poorer QOL due to a higher burden of unaddressed geriatric syndromes and medical comorbidities, resulting from limited access to relevant healthcare services [67]. As this was a cross‐sectional analysis, causality cannot be established. Also, we did not assess the association between QOL and the cumulative burden of medical comorbidities. Despite these limitations, this multisite study involving 400 PLH ≥50 years provides an important starting point for understanding the burden of comorbidities and their impact on QOL. This study also highlights the need for tailored interventions to better identify, prevent and address the ageing‐related needs of this population.

## 6 Conclusions

In summary, we observed good overall perceived QOL among older PLH in urban Tanzania, despite the high burden of geriatric syndromes and medical comorbidities. Pre‐frailty, functional impairment and depression were significantly associated with poorer QOL. These findings suggest a level of resilience within this population but also highlight the need for holistic care that includes screening and management of age‐related comorbidities. Additional research is needed to explore how these services can be effectively and sustainably implemented within HIV clinics without compromising the quality of HIV care.

## Author Contributions

**Theresia A. Ottaru**: conceptualization; data curation; formal analysis; funding acquisition; methodology; project administration; validation; visualization; writing – original draft preparation. **Christopher Mbotwa**: data curation; formal analysis; validation; visualization; writing – review and editing. **Dannielle Grayer**: writing – review and editing. **Joan Rugemalila**: writing – review and editing. **Lisa R. Hirschhorn**: conceptualization; funding acquisition; methodology; supervision; writing – review and editing. **Sylvia Kaaya**: conceptualization; funding acquisition; methodology; supervision; writing – review and editing. **Grayson Nyamsogoro**: investigation; writing – reviewing and editing. **Rosemary Kiboma**: investigation; writing – reviewing and editing. **Herieth Mboya**: investigation; writing – reviewing and editing. **Irene Mageni**: investigation; writing – reviewing and editing. **Pilly Chillo**: writing – review and editing. **Edith Tarimo**: writing – review and editing. **Mary Clare Masters**: writing – review and editing. **Bethann Conover**: project administration; writing – editing and reviewing. **Claudia Hawkins**: conceptualization; funding acquisition; methodology; supervision; writing – review and editing. All authors have approved the final version and agreed to its submission.

## Funding

This research was funded by the Fogarty International Center of the National Institutes of Health through a supplemental award for D43 grant number D43TW010946. The study was also supported by a Robert J. Havey, MD Project Award, Institute for Global Health, Northwestern University.

## Conflicts of Interest

The authors declared no potential conflicts of interest with respect to the research, authorship and/or publication of this article.

## Disclaimer

The content is solely the responsibility of the authors and does not necessarily represent the official views of the National Institutes of Health.

## Supporting information




**Supporting File 1**: jia270163‐sup‐0001‐TableS1.docx

## Data Availability

The dataset analysed during the current study is available from the corresponding author on reasonable request.
