# Supplementary material for: Correlates of Poor Quality of Life Among Older People Living With HIV: A Cross‐Sectional Analysis of the Tanzania HIV and Aging Cohort Study
Source: J Int AIDS Soc. 2026 Jul 17;29(7):e70163. doi: 10.1002/jia2.70163 (PMC13376838; doi:10.1002/jia2.70163)
Supplement: Supplementary file 1 — Supporting File 1: jia270163‐sup‐0001‐TableS1.docx [file JIA2-29-e70163-s001.docx]

**Supplementary table 1:** SupplementaryMultivariate regression analyses for medical comorbidities and geriatric syndromes associated with QOL domains

| **Variables/Domains** | **Physical** | **Independence** | **Social** | **Environmental** | **Spirituality** | **Psychological^₱^** |
| --- | --- | --- | --- | --- | --- | --- |
| **Geriatric conditions** |  |  |  |  |  |  |
| Frailty |  |  |  |  |  |  |
| No frail | Ref | Ref | Ref | Ref | Ref | Ref |
| Pre-frail | **-4.51(-7.82, -1.20)^**^** | **-3.48(-6.66, -.30)^*^** | -2.82(-6.71, 1.07) | -2.93(-6.77, .91) | -2.00(-5.86, 1.86) | **-5.04(-9.15, -.92)^*^** |
| Frail | **-6.33(-12.16, -.51)^*^** | **-11.97(-17.57, -6.37)^***^** | .37(-6.48, 7.23) | **7.11(.35, 13.88)^*^** | -1.02(-7.82, 5.77) | -1.37(-8.63, 5.89) |
| Cognition |  |  |  |  |  |  |
| No cognitive impairment | Ref | Ref | Ref | Ref | Ref | Ref |
| Mild cognitive impairment/Dementia | -3.95(-10.06, 2.16) | .66(-5.22, 6.54) | 3.01(-4.18, 10.20) | -1.29(-8.39, 5.81) | .93(-6.20, 8.06) | 3.98(-3.63, 11.58) |
| Number of ADL difficulties |  |  |  |  |  |  |
| None | Ref | Ref | Ref | Ref | Ref | Ref |
| 1-3 | **-12.80(-16.40, -9.21)^***^** | **-4.08(-7.54, -.62)^*^** | **-5.83(-10.06, -1.60)^**^** | **-5.77(-9.95, -1.60)^**^** | -1.43(-5.62, 2.77) | **-9.19(-13.64, -4.74)^***^** |
| 4-6 | **-17.05(-22.81, -11.28)^***^** | -5.18(-10.72, .37) | -6.58(-13.36, .20) | **-10.07(-16.77, -3.37)^**^** | -.72(-7.45, 6.00) | **-16.96(-24.00, -9.93)^***^** |
| >6 | **-22.99(-31.14, -14.83)^***^** | **-17.73(-25.58, -9.89)^***^** | **-16.95(-26.55, -7.35)^***^** | **-12.67(-22.15, -3.20)^**^** | 3.28(-6.24, 12.80) | **-27.39(-37.17, -17.61)^***^** |
| At risk of social isolation | .78(-2.69, 4.24) | 2.81(-.52, 6.15) | -1.00(-5.09, 3.08) | -1.00(-5.03, 3.03) | -2.99(-7.04, 1.05) | -2.02(-6.27, 2.23) |
| Depression (PHQ-9) |  |  |  |  |  |  |
| None or minimal (0-4) | Ref | Ref | Ref | Ref | Ref | - |
| Mild (5-9) | **-7.17(-12.03, -2.31)^**^** | -2.46(-7.13, 2.21) | -4.15(-9.87, 1.56) | -4.87(-10.51, .77) | **-14.43(-20.10, -8.77) ^***^** | - |
| Moderate/Moderately severe (10-19) | **-24.19(-32.22, -16.15)^***^** | **-12.40(-20.11, -4.67)^**^** | **-12.09(-21.55, -2.64)^**^** | **-16.26(-25.59, -6.93)^***^** | **-21.33(-30.70, -11.96)^***^** | - |
| **Medical comorbidities** |  |  |  |  |  |  |
| Hypertension ^‡^ | .04(-3.09, 3.16) | -.89(-3.89, 2.11) | -.42(-4.10, 3.25) | -1.35(-4.98, 2.28) | **-5.43(-9.07, -1.78)^**^** | -1.10(-4.98, 2.79) |
| Diabetes | .53(-4.07, 5.13) | 1.93(-2.50, 6.35) | 1.33(-4.08, 6.74) | 2.17(-3.18, 7.51) | 4.18(-1.19, 9.55) | 1.55(-4.19, 7.28) |
| Renal dysfunction ^¶^ | -2.14(-6.15, 1.87) | .28(-3.58, 4.14) | 1.13(-3.59, 5.85) | 2.25(-2.41, 6.91) | .10(-4.58, 4.78) | 1.76(-3.24, 6.77) |
| Dyslipidaemia ^§^ | -2.19(-5.99, 1.61) | -.23(-3.88, 3.43) | -2.88(-7.35, 1.59) | -2.68(-7.10, 1.73) | -.74(-5.18, 3.69) | 1.17(-3.56, 5.90) |
| Number of medications used |  |  |  |  |  |  |
| 0-1 | Ref | Ref | Ref | Ref | Ref | Ref |
| ≥2 | -2.68(-7.75, 2.40) | **-6.43(-11.31, -1.55)^**^** | -1.13(-7.10, 4.84) | -2.43(-8.32, 3.47) | -4.22(-10.14, 1.70) | **-6.66(-12.95, -.37)^*^** |
| BMI (kg/m²) |  |  |  |  |  |  |
| Normal | Ref | Ref | Ref | Ref | Ref | Ref |
| Underweight | -4.26(-10.51, 1.99) | -5.91(-11.93, .10) | -1.05(-8.41, 6.31) | **-9.34(-16.61, -2.08)^*^** | 3.99(-3.31, 11.29) | -3.20(-10.92, 4.53) |
| Overweight | .34(-3.15, 3.83) | -2.10(-5.46, 1.26) | **-4.12(-8.23, -.02)^*^** | .58(-3.47, 4.64) | .17(-3.90, 4.25) | -.73(-5.08, 3.62) |
| Obese | -.09(-4.11, 3.92) | -2.72(-6.58, 1.14) | -.91(-5.64, 3.81) | -2.77(-7.43, 1.89) | 2.91(-1.78, 7.59) | -1.45(-6.47, 3.57) |
| **Adjusted confounders** |  |  |  |  |  |  |
| Sex |  |  |  |  |  |  |
| Female | Ref | Ref | Ref | Ref | Ref | Ref |
| Male | -3.59(-7.2, .060) | -1.49(-5.00, 2.02) | -3.79(-8.09, .50) | 1.08(-3.16, 5.32) | -.22(-4.48, 4.04) | -1.50(-5.54, 2.55) |
| Interaction between sex and depression |  |  |  |  |  |  |
| Female*None or minimal depression |  | Ref | Ref | Ref | Ref | Ref |
| Male*Mild depression | .26(-7.05, 7.57) | 1.89(-5.13, 8.91) | -4.41(-13.0, 4.18) | -6.59(-15.07, 1.89) | 1.21(-7.31, 9.72) |  |
| Male*moderate/Moderately severe depression | **14.72(4.08, 25.37) ^**^** | 9.57(-.66, 19.80) | -.98(-13.50, 11.54) | 3.98(-8.37, 16.35) | 3.37(-9.03 15.78) |  |
| Age (in years) | **.29(.053, .52) ^*^** | -.001(-.23, .22) | .11(-.16, .39) | .22(-.05, .49) | **.29(.01, .55) ^*^** | .22(-.07, .51) |
| Education level |  |  |  |  |  |  |
| No formal education | Ref | Ref | Ref | Ref | Ref | Ref |
| Primary | .07(-4.34, 4.48) | 2.72(-1.52, 6.95) | -3.49(-8.68, 1.69) | -.95(-6.07, 4.17) | **.28(.01, .55) ^*^** | -1.97(-7.45, 3.50) |
| Secondary+ | -1.02(-6.61, 4.57) | .01(-5.35, 5.38) | -5.06(-11.65, 1.50) | 2.06(-4.42, 8.55) | -.42(-5.57, 4.72) | -2.64(-9.60, 4.32) |
| Estimated monthly income (TZS) |  |  |  |  |  |  |
| < 100,000 | Ref | Ref | Ref | Ref | Ref | Ref |
| 100,000-500,000 | .64(-2.62, 3.91) | 1.06(-2.08, 4.20) | 3.10(-.74, 6.94) | **5.47(1.68, 9.26) ^**^** | 2.80(-1.01, 6.61) | -1.14(-5.22, 2.94) |
| >500,000 | 5.22(-.78, 11.23) | 2.03(-3.74, 7.79) | **7.45(.39, 14.51) ^*^** | **15.88(8.91, 22.85) ^***^** | **7.66(.66, 14.66) ^*^** | 4.97(-2.54, 12.47) |
| Has Health insurance | **-5.55(-9.52, -1.59) ^**^** | -.89(-4.70, 2.92) | 2.23(-2.43, 6.89) | **9.19(4.59, 13.79) ^***^** | 2.54(-2.07, 7.17) | 3.61(-1.30, 8.52) |
| Duration since in ART |  |  |  |  |  |  |
| <10 | Ref | Ref | Ref | Ref | Ref | Ref |
| 10-19 | .14(-2.77, 3.06) | 2.03(-.77, 4.83) | -1.79(-5.22, 1.64) | -2.30(-5.68, 1.09) | -1.05(-4.45, 2.35) | -2.37(-6.01, 1.27) |
| ≥20 | .51(-8.90, 9.92) | **-10.55(-19.59, -1.51) ^*^** | 3.71(-7.35, 14.79) | -3.70 (-14.63, 7.22) | 7.69(-3.28, 18.66) | -2.91(-14.60, 8.78) |
| Undetectable viral load (< 50 copies/ml) | 1.09(-2.41, 4.58) | -.52(-3.87, 2.84) | -.13(-4.24, 3.98) | -1.39(-5.45, 2.66) | -1.65(-5.7, 2.42) | -2.03(-6.38, 2.32) |

^‡^ Hypertension: SBP≥140 mmHg or DBP≥90 mmHg or on antihypertensive medication

^§^ Diabetes: FBG≥7 mmol/L or on antidiabetic medication

^§^ Dyslipidaemia: At least one of these: LDL cholesterol: > 2.6 mmol/L or Triglycerides: > 1.7 mmol/L or Total cholesterol: > 5.2 mmol/L or HDL: < 1 mmol/l regardless of sex

^¶^ Renal dysfunction: eGFR<60 ml/min/1.73m^2^

^*^ p-value <0.05, ^**^ p-value <0.01, ^***^ p-value <0.001

^₱^ Variable depression excluded from the analysis

In domain specific multivariable analysis, adjusting for age, sex, education level, income, health insurance, duration on ART, and viral load, several geriatrics conditions, hypertension, medication use and age were associated with QOL.

Individuals in pre-frail and frail categories had lower scores in physical and independence domains, while individuals in the pre-frail but not frail category had lower psychological domain scores. Impaired ADLs showed a dose–response relationship with physical, environmental and psychological domains with progressively greater score reductions observed as the number of difficulties increased. Individuals with 1-3 and >6 ADL difficulties also showed significant score reductions in independence and social domains.

Moderate/moderately severe depression was associated with lower QOL scores in all QOL domains. Mild depression was also associated with lower scores in physical and spiritual domains. Individuals on more than two medication had low scores in independence and psychological domains. Higher income was significantly associated with environmental domain scores. Compared with the <100,000/- income group, individuals in the 100,000/- to 500,000/- and >500,000/- categories had significantly higher environmental domain scores (β = 5.47, 95% CI: 1.68–9.26 and β = 15.88, 95% CI: 8.91–22.85, respectively). Similarly, those with higher income had higher scores in social and spirituality domains while having health insurance was associated with significant higher score in the environmental domain.
